# Interdisciplinary Periprocedural Management of Patients Undergoing Transapical TMVI with the Tendyne System: A Narrative Review and Institutional Experience

**DOI:** 10.1093/icvts/ivaf181

**Published:** 2025-08-14

**Authors:** Cyril D Ferro, Fabien Praz, Nicolas Brugger, David Reineke, Sandra Terbeck, Florian Setzer, Stephan Windecker, Gabor Erdoes

**Affiliations:** Department of Cardiology, Inselspital, Bern University Hospital, University of Bern, 3010 Bern, Switzerland; Department of Cardiology, Inselspital, Bern University Hospital, University of Bern, 3010 Bern, Switzerland; Department of Cardiology, Inselspital, Bern University Hospital, University of Bern, 3010 Bern, Switzerland; Department of Cardiac Surgery, Inselspital, Bern University Hospital, University of Bern, 3010 Bern, Switzerland; Department of Anesthesiology and Pain Medicine, Inselspital, Bern University Hospital, University of Bern, 3010 Bern, Switzerland; Department of Cardiac Surgery, Inselspital, Bern University Hospital, University of Bern, 3010 Bern, Switzerland; Department of Cardiology, Inselspital, Bern University Hospital, University of Bern, 3010 Bern, Switzerland; Department of Anesthesiology and Pain Medicine, Inselspital, Bern University Hospital, University of Bern, 3010 Bern, Switzerland

**Keywords:** mitral regurgitation, mitral stenosis, transcatheter edge-to-edge repair, transcatheter mitral valve replacement, transcatheter mitral valve implantation

## Abstract

**Objectives:**

Mitral regurgitation (MR) represents the most common valvular heart disease (VHD) in the Western world. While transcatheter mitral valve repair (M-TEER) is the leading interventional treatment for surgically high-risk patients, transcatheter mitral valve implantation (TMVI) is reserved for selected patients with unsuitable anatomy for M-TEER. This review aims to summarize our institutional experience with transapical TMVI using the Tendyne valve (Abbott Vascular, CA, USA), focusing on interdisciplinary preoperative, intraoperative, and postoperative management strategies.

**Methods:**

We conducted a narrative review of current literature on TMVI with the Tendyne system and integrated it with a comprehensive analysis of our interdisciplinary clinical experience. Data were collected regarding patient selection, imaging protocols, procedural techniques, and postoperative care.

**Results:**

Utilizing the Tendyne valve, TMVI addresses symptomatic moderate-to-severe or severe MR in patients unsuitable for conventional surgery or M-TEER. Successful outcomes require thorough patient selection, including assessment of mitral annular calcification, absence of intracardiac thrombus, low left ventricular outflow tract (LVOT) obstruction risk, and optimal annular sizing. Multimodal imaging, particularly transoesophageal echocardiography and cardiac computed tomography, is essential for procedural planning and execution. TMVI is performed under general anaesthesia with intraoperative transoesophageal guidance and haemodynamic monitoring to minimize complications such as LVOT obstruction, bleeding, and valve malposition. Postoperative management emphasizes haemodynamic stabilization, bleeding control, and surveillance for paravalvular leaks or device dysfunction.

**Conclusions:**

TMVI with the Tendyne valve provides a viable and effective treatment for selected patients with symptomatic relevant MR. Optimal outcomes are dependent on meticulous interdisciplinary collaboration, advanced imaging protocols, and comprehensive perioperative management.

## Graphical Abstract

**Figure:**
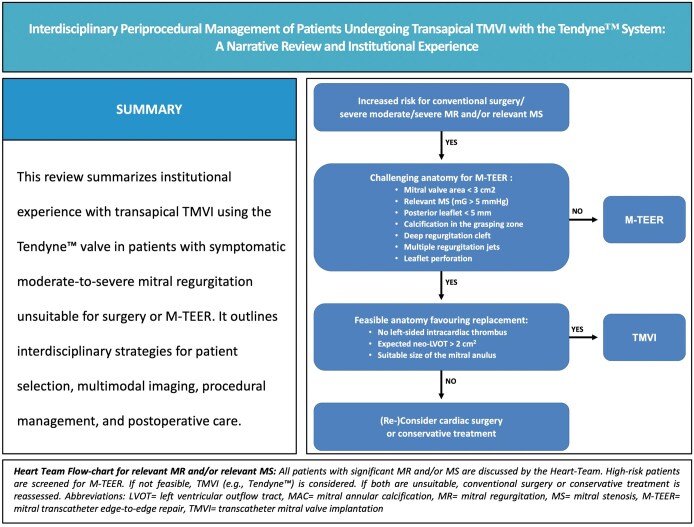

## INTRODUCTION

Mitral regurgitation (MR) is the most frequent valvular heart disease (VHD) lesion in the Western World.^1^ During decades, surgery was the only treatment option, but recently minimally-invasive treatment of the mitral valve (MV) have been developed. While transcatheter repair is currently the most widely used technique, transcatheter mitral valve implantation (TMVI) is only applied in selected patients with unsuitable anatomy or contraindications for mitral transcatheter edge-to-edge repair (M-TEER). This review summarizes the current literature and our TMVI experience^2^ using the Tendyne valve (Abbott Vascular, CA, USA) focusing on periprocedural management.

## SCREENING FOR TMVI

**Figure 1.**
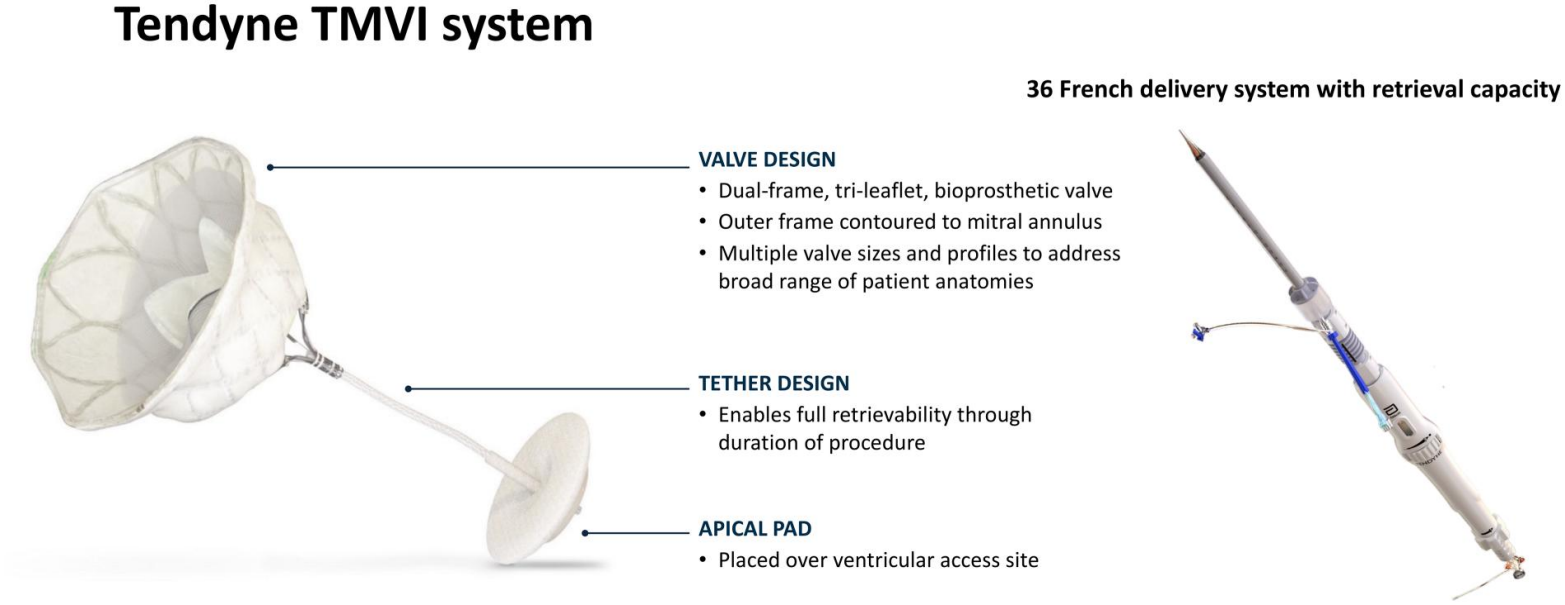
The Tendyne TMVI System. The Tendyne device is a trileaflet bioprosthesis valve consisting of a nitinol self-expanding stent frame, which is covered with porcine pericardium. The device is designed to be implanted into a beating heart through a transapical approach via a left minithoracotomy. The delivery system enables full repositioning and retrieval of the valve

**Figure 2.**
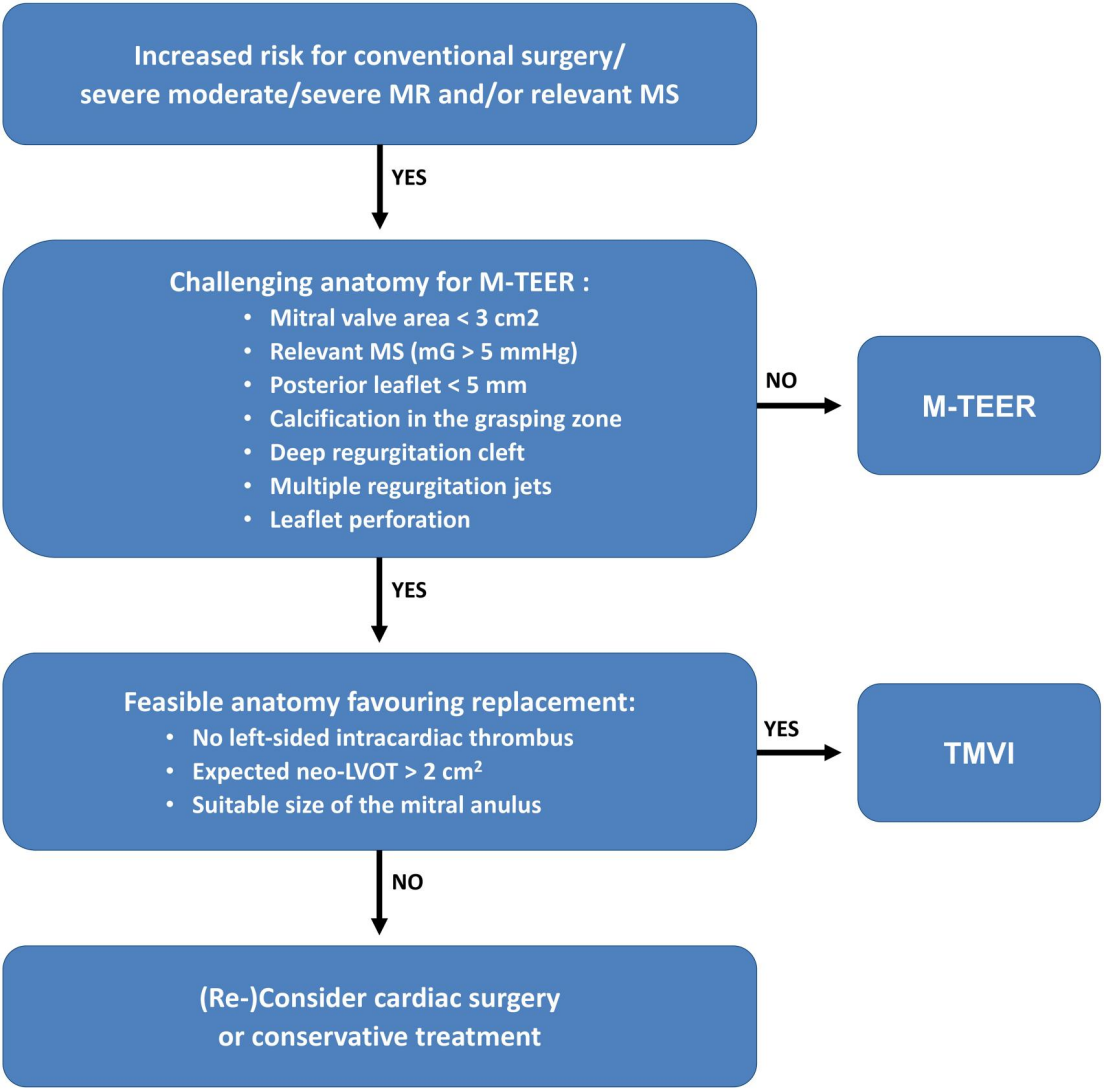
Heart Team Flow-Chart for Relevant MR and/or relevant MS. All patients with moderate-to-severe, severe MR, and/or relevant MS are discussed in our interdisciplinary Heart-Team. Patients with an increased risk for conventional surgery are further screened for a transcatheter approach with M-TEER. In cases in which M-TEER is not feasible, patients are evaluated for TMVI devices (including the Tendyne valve). In cases in which neither M-TEER nor TMVI are feasible, reconsideration for conventional surgery (accepting an increased risk) or for a conservative treatment is indicated. Abbreviations: LVOT, left ventricular outflow tract; MAC, mitral annular calcification; MR, mitral regurgitation; MS, mitral stenosis; M-TEER, mitral transcatheter edge-to-edge repair; TMVI, transcatheter mitral valve implantation

All patients with relevant symptomatic MR are evaluated and discussed within the interdisciplinary Heart Team. Patients who are high risk for conventional surgical treatment or have complex anatomy for M-TEER are screened for TMVI with the Tendyne valve.^3^ The Tendyne device is a biological valve prosthesis designed to be implanted into a beating heart through transapical access via a left minithoracotomy. The valve consists of a nitinol self-expanding stent frame, which is covered with porcine pericardium (**Figure 1**). Screening failures for TMVI are common with rates reported up to 60%.^4^ Limiting factors consist of both anatomical parameters (size of the native MV, the estimated risk for left ventricle outflow tract [LVOT] obstruction, severe mitral annular or leaflet calcification and intracardiac left-sided thrombus) and comorbidities (concomitant conditions with an expected survival <1 year or conditions in which a significant improvement in quality of life through the intervention is unlikely)^5^ (**Figure 2**). Of note, severe frailty represents an important limitation since candidates should be able to safely undergo left thoracotomy. Further challenges for TMVI include annular size variation during the cardiac cycle (the mitral annular area increases more than 20% in diastole),^4^ the heterogeneity of the pathology, and the complexity of the MV anatomy.^4^^,^^6^^,^^7^

**Figure 3.**
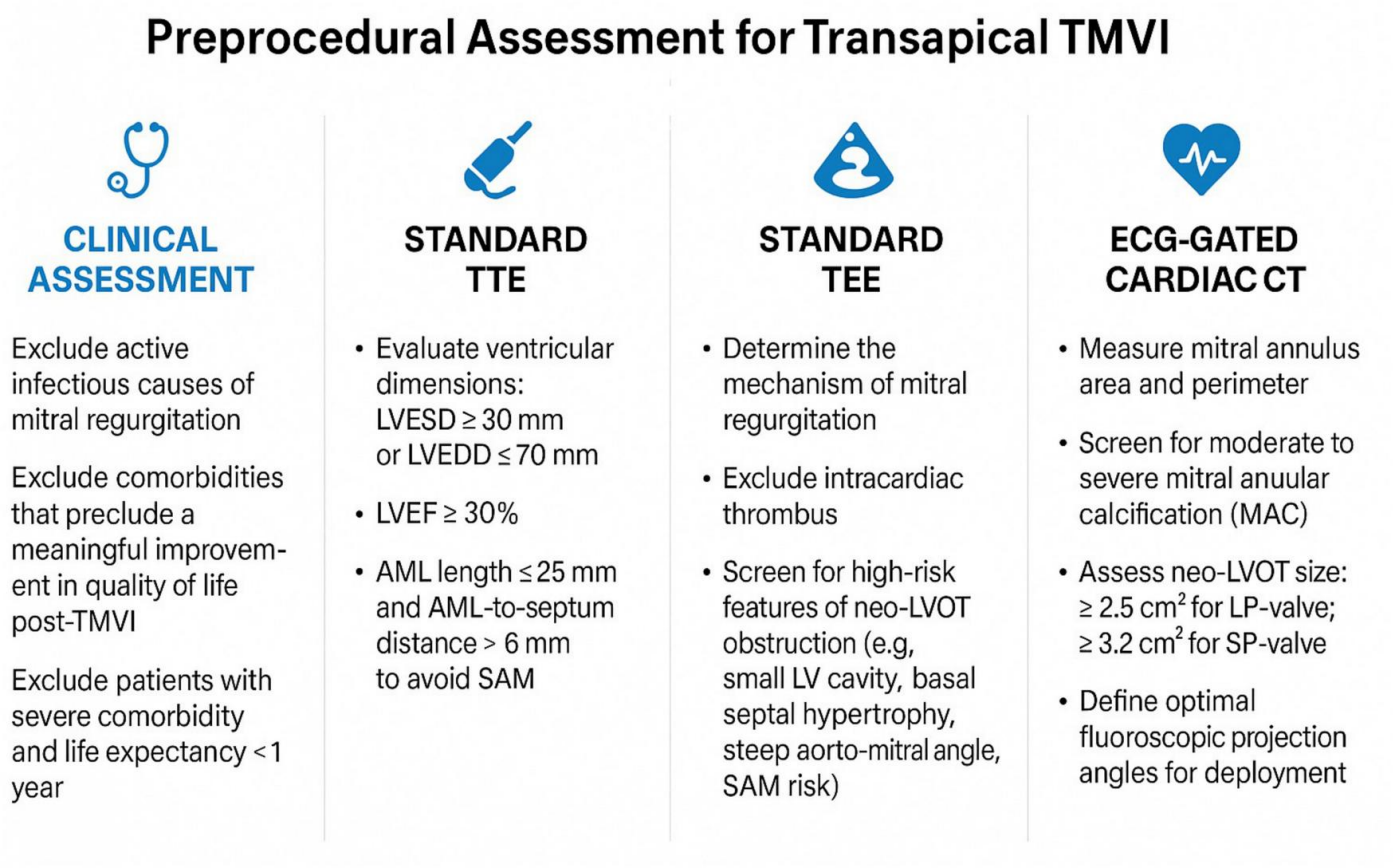
Routine Screening Steps of Candidates for Transapical TMVI. Hard exclusion criteria include the mitral annulus size, the minimal size of the neo-LVOT and active endocarditis. Abbreviations: AML, anterior mitral leaflet; LVEDD, left ventricular end diastolic diameter; LVEF, left ventricular ejection fraction; LVOT, left ventricular outflow tract; MAC, mitral annular calcification; MR, mitral regurgitation; SAM, systolic anterior motion; TMVI, transcatheter mitral valve implantation

Transthoracic echocardiography (TTE) remains the first-line imaging modality to assess MR severity and mechanism during screening of TMVI candidates. Transoesophageal echocardiography (TEE) allows to more precisely define MV anatomy and better evaluate the feasibility of M-TEER or TMVI, respectively. Patients not suitable for M-TEER are systematically considered for TMVI, in particular those with calcifications in the grasping zone, a posterior MV leaflet <5 mm, leaflet perforation or regurgitant cleft, small MV area (<3.0 cm^2^), and/or an increased baseline MV gradient due to rheumatic mitral stenosis or mitral annular calcification.^8^ Important inclusion criteria for TMVI with a Tendyne device are the absence of intracardiac thrombus, a suitable size of the MV (there are 13 sizes of Tendyne valves in total, covering an annular perimeter range from 130 up to 150 mm) and a proper distance from the MV to the LVOT (**Figure 3**).

In addition to echocardiography, right and left heart catheterization are performed, to confirm MR severity, measure pulmonary artery pressures, and exclude concomitant coronary artery disease. Cardiac magnetic resonance imaging (cMRI) is not routinely used, but may have a supportive role, when other imaging modalities are inconclusive regarding MR severity or left ventricular dimensions. In addition, identification of scar tissue by cMRI may have a prognostic value as demonstrated for patients undergoing surgical valve replacement.^9^ Multislice cardiac computed tomography (CCT) is a standard tool for anatomical screening and TMVI planning. CCT acquisition should include the entire cardiac cycle to record the dynamic changes in the configuration of the LVOT, the left ventricle (LV) and the MV during systole and diastole. The evaluation of the MV annulus size is performed just before the atrial contraction at 85% to 95% of the cardiac cycle during mid to late diastole.

**Figure 4.**
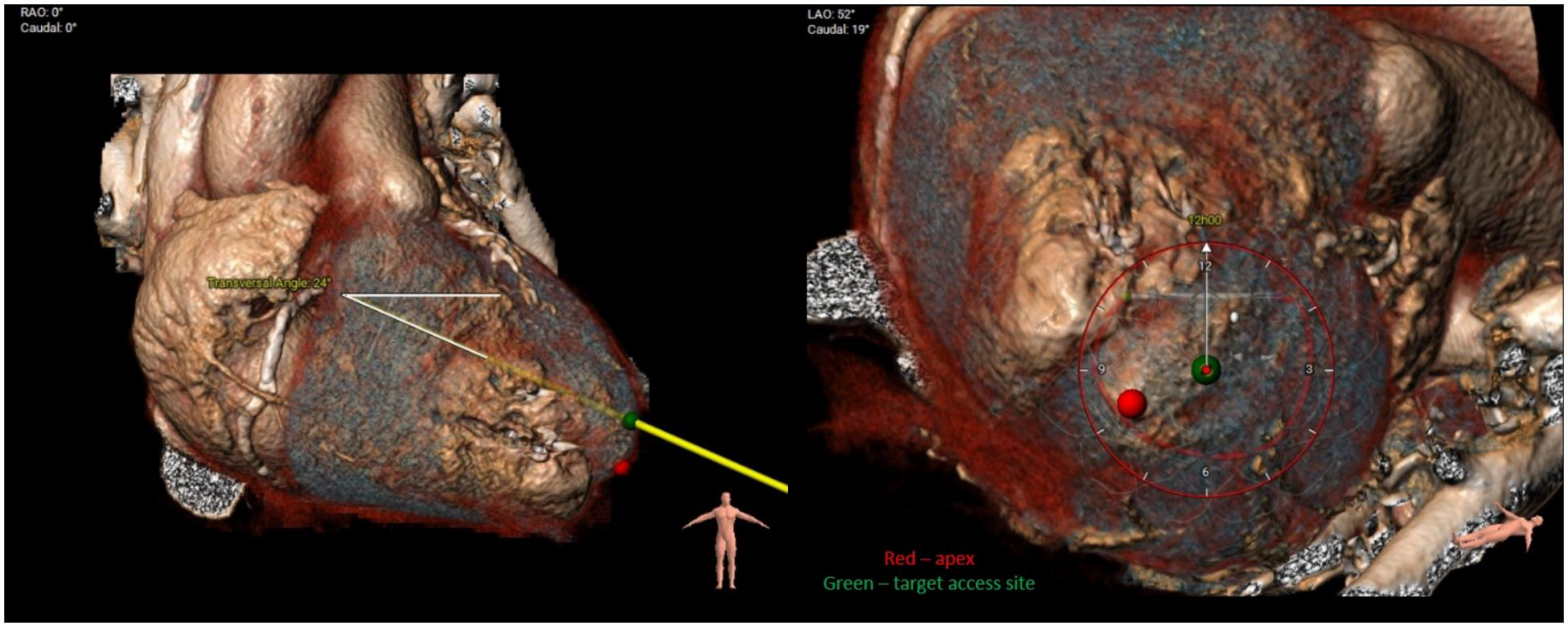
Simulation of Transapical Access Based on CT Images. (A) The yellow line indicates the calculated access perpendicular to the mitral annulus. (B) The clock indicates the anticipated catheter rotation for optimal valve orientation (in this 12 o’clock). The red dot indicates the true apex of the heart and the green dot the optimal access point to achieve perpendicularity to the mitral annulus (or optimize neo-LVOT size)

The use of 3-dimensional (3D) reconstruction software is helpful to assess the size and geometry of the annulus and to provide precise information on the landing zone for the Tendyne prosthesis. The implantation of the Tendyne device leads to the formation of a neo-LVOT, which is the smallest residual space between the device and the interventricular septum. This neo-LVOT should have an area of at least 2 cm^2^.^4^^,^^10^ The definitive cut-off value of the neo-LVOT for a screening failure may vary and depends on the model and size of the device and the individual cut-off values of each company facilitating those devices. In the 3D valve simulation, anatomical high-risk features for LVOT obstruction include basal septal hypertrophy, small LV cavities, and large aorto-mitral angulation. The localization and density of mitral annular calcification are precisely evaluated using CCT because of the risk of relevant paravalvular leak (PVL) or valve underexpansion. Finally, CCT defines the fluoroscopic projection angles to be used during the intervention (**Figure 4**).

## PATIENT PREPARATION AND ANAESTHESIOLOGICAL CONSIDERATIONS

The patient cohort represents a high-risk surgical population characterized by numerous comorbidities. All individuals undergo preoperative evaluation, including a thorough cardiac assessment as well as standardized evaluations of quality of life and physical performance status. The pre-anaesthetic assessment focuses primarily on physical examination, with specific attention to airway management, vascular access, and anatomical considerations relevant to the feasibility of regional anaesthesia techniques. These assessments are conducted in accordance with the guidelines established by the European Society of Anaesthesiology and Intensive Care (ESAIC), the American Society of Anesthesiologists (ASA), and the European Society of Cardiology (ESC).^11–13^

A comprehensive review of the patient’s current pharmacologic regimen is undertaken, with most medications continued throughout the perioperative period. Adjustments are individualized, particularly concerning antihypertensive and antidiabetic therapies. Acetylsalicylic acid is routinely maintained, while other anticoagulants, such as vitamin K antagonists, are discontinued in line with procedural bleeding risk. Adequate time is allocated for preoperative optimization in accordance with enhanced recovery after (cardiac) surgery protocols (ERAS [Enhanced recovery after surgery] and ERACS [Enhanced recovery after cardiac surgery]).^14^^,^^15^

TMVI procedures with Tendyne are conducted under general anaesthesia. Prior to induction, an arterial line is inserted. The complete anaesthetic setup includes large-bore peripheral intravenous access and central venous access, comprising a triple-lumen catheter for drug administration and central venous pressure monitoring, along with a volume access line capable of rapid transfusion and accommodating either a transvenous pacer or a pulmonary artery catheter. The use of haemodynamic monitoring is recommended for managing intraprocedural fluid and vasopressor therapy (**Table 1**). Drawing on institutional experience with transapical procedures such as transapical transcatheter aortic valve implantation (TA-TAVI) and minimally invasive cardiac surgery-mitral valve repair (MICS-MVR), all patients receive an erector spinae plane block (ESPB) at the level of surgical access.^16^^,^^17^ ESPB is a paraspinal fascial plane block where a local anaesthetic is administered to block the dorsal and ventral rami of the thoracic spinal nerves. For the block, we use the maximum weight-based dosage for a single shot of 0.75% ropivacaine, mixed with dexmedetomidine to extend the duration of the effect. Intraoperatively, all patients undergo multimodal neuromonitoring, including near-infrared spectroscopy (NIRS) and processed electroencephalography (pEEG), to ensure cerebral perfusion and anaesthetic depth. In uncomplicated cases, ultra fast-track, on-table extubation is typically performed immediately after the procedure. Haemodynamically stable patients are transferred to the intermediate care unit, while those requiring high-dose vasopressor support are managed in the intensive care unit.

**Table 1.** Green Zone, Red Flags and Associated Diagnostic/Therapeutic Options

Parameter	Green zone	Red flags	Diagnostic/therapeutic options
Vasopressor dose	norepinephrine ≤ 0.1 mcg/kg/min, dose reduction possible	Escalating doses, necessity to restart after cessation	TTE, clinical judgement, administer fluid bolus
Lactate clearance	≤ 2 mmol/l	No lactate clearance, rising lactate plasma levels	TTE, high clinical suspicion for malperfusion and or abdominal complications, administer fluid bolus
Drainage (hourly)	< 100 ml/hr (“first portion” after admission may not be included), character of drain fluid (haematocrit?)	> 200 ml/hr or rising amount of drainage. (Acquired) coagulopathy? TAH?	Thromboelastometry, blood count or standard coagulation tests. Surgical control of Haemorrhage necessary?
Skin temperature (central vs peripheral) and mottling score	< 5°C difference and/or falling delta plus normothermia, mottling score ≤ 2	Rising delta temperature, mottling score ≥ 3 or increasing	If other signs of shock: TTE, Evaluation of circulatory parameters
Urine output	> 0.5 ml/kg/hr	< 0.5 ml/kg/hr or decreasing urine output	Evaluate fluid bolus, check other circulatory parameters, echocardiography

Patients with isolated severe MR often experience improvement in severity of MR after induction of anaesthesia due to a reduction in peripheral vascular resistance. For patients with pulmonary arterial hypertension, low-dose vasopressin has proven effective when the peripheral resistance is profoundly reduced by anaesthetics, as it does not affect the pulmonary resistance. This helps to maintain adequate myocardial perfusion, especially in patients with concomitant coronary artery disease, aortic stenosis, or cerebrovascular disease. The implantation of the TMVI valve poses a unique challenge for anaesthetic management (**Table 1**). The primary focus is on maintaining adequate preload and preventing excessive tachycardia or LVOT obstruction of varying degrees during the final procedural step. To treat hypotension, volume administration is crucial to ensure sufficient preload, while vasopressors such as norepinephrine or vasopressin can be utilized to stabilize blood pressure without increasing myocardial oxygen demand. Positive inotropic agents are generally avoided, as they may exacerbate outflow tract obstruction. Haemodynamic monitoring and using central venous pressure, arterial line, and transoesophageal echocardiography allows for precise titration of fluids and vasopressors.

## SPECIFIC SURGICAL CONSIDERATIONS TO OPTIMIZE TRANSAPICAL ACCESS

### Preprocedural planning

Optimal transapical access is critical to the success of TMVI with the Tendyne system. Patient positioning should replicate that of preprocedural CCT to ensure accurate anatomical alignment and to avoid distortion of pre-planned trajectories. CCT aids in identifying the most suitable intercostal space for apical access by evaluating proximity to the apex, avoidance of major coronary vessels, trajectory relative to the MV plane, and risk of left ventricular outflow tract (LVOT) obstruction. In some cases, a slightly posterior access angle is selected to direct the valve away from the interventricular septum and optimize LVOT patency.

### Intraoperative confirmation

The planned access route and apex location should be confirmed by transthoracic echocardiography (TTE) prior to incision. In patients with prior thoracic surgery, anatomical variations or adhesions may require modified approaches, although one-lung ventilation is rarely necessary. Before entering the pericardium, apex palpation is performed to determine the optimal point of access as simulated by CCT (poke test), which needs to be confirmed by TEE (see also the section on TEE guiding).

### Safe access technique

Achieving a perpendicular trajectory to the MV is important for optimal valve placement and to facilitate apical haemostasis at the end of the procedure. Following opening of the pericardium, stay sutures can be placed if they facilitate access, especially caudally. To correct for a too anterior position, 1 or 2 cloths should be carefully placed under the heart with strong sutures attached to them to ensure safe removal. Two concentric 3-0 Prolene purse-string sutures, reinforced by Teflon pledgets, are then placed at the LV apex around the anticipated puncture site to ensure haemostasis. Sutures should be placed deeply and bleeding may occur, confirming adequate tissue depth. Visible coronary arteries should, if possible, be avoided. The curve of the needle should be followed to prevent laceration of the apex and the Teflon felts positioned as a full circle around the puncture site. Sutures should not be anchored in fatty tissue.

### Valve delivery

The LV is then punctured with an angiographic needle and the MV crossed with a standard guidewire. An 8-French sheath is introduced over the wire and de-aired followed by introduction of a 7-French Swan-Ganz catheter, which is placed into the left atrium (LA). Under intermittent balloon inflation and TEE guidance, the balloon is advanced through the MV to exclude entrapment in the subvalvular apparatus. If smooth balloon flossing is hindered, entrapment should be suspected and guidewire placement corrected. The 36-French Tendyne delivery system is then advanced through the LV apex directly over the standard wire in a sheathless fashion. In pre-operated patients, the Tendyne system usually penetrates the apex without difficulty due to the stability of the surface. In a virgin chest, the apex tends to be mobile and insertion often provokes haemodynamically relevant invagination of the heart. This can be overcome or avoided by rotating the sheath or by stepwise predilatation. Should this not bring the desired effect, frank incision of the puncture site with an 11 blade can be considered.

### Bleeding management and closure

Perpendicularity of the delivery system and tether are essential to minimize bleeding throughout the procedure and prevent apex laceration. Further haemostasis is achieved by pad application and locking at the entry point. A large pad should be preferred whenever possible and always considered for low profile valves, as it will avoid myocardial invagination and prevent atrial valve migration. Bleeding at this stage can be controlled through heparin antagonization with protamine, local compression (taking care not to loosen the tie and dislocating the valve) and additional sutures, always reinforced with felt pledgets. Excessive bleeding may lead to LVOT obstruction and further compromise patient’s haemodynamics. Before final tether cutting, it is essential to ensure that no intrathoracic materials (e.g., support gauze or retraction devices) interfere with heart position or tether tension. A residual tether length should be preserved to allow for re-tensioning in select cases, although this is rarely required (<2%).^18^ A pleural drain should be placed. The pericardium may be left open, and the ribs should be sutured together, as a pulmonary hernia can also occur on the left side.

## PROCEDURAL STEPS AND ECHOCARDIOGRAPHIC GUIDING OF TRANSAPICAL TMVI

After exposure of the cardiac apex, transapical puncture is guided using bi-plane echocardiography to obtain bi-commissural and 3-cavity (LVOT) views. The surgeon palpates the apex (“poke test”), and the indentation is confirmed on both views to match the pre-defined trajectory from cardiac CT, which often differs from the anatomical apex.

The implant catheter is then advanced over the guidewire and positioned 1-2 cm above the mitral annulus, with alignment optimized in the bi-commissural and 3-cavity views. Valve deployment is performed under continuous bi-plane and 3D echocardiographic monitoring. Rotational orientation is confirmed using key visual markers—2 short elements in the bi-commissural view and asymmetric “rabbit ears” in the LVOT view—intended to align with the aorto-mitral curtain. In the 3D surgical view (aorta at 12 o’clock), counterclockwise rotation of the image corresponds to clockwise rotation of the catheter due to the transapical approach.

Once the prosthesis has been deployed, the valve is assessed using colour Doppler from 0 to 180° to detect PVLs and, if necessary, an increase in traction on the tensioner can be applied as a corrective manoeuvre. In the 3-cavity view and in the apical trans-gastric view (5-cavity equivalent), the patency of the neo-LVOT is assessed and the tension reduced in the event of significant obstruction.

**Figure 5.**
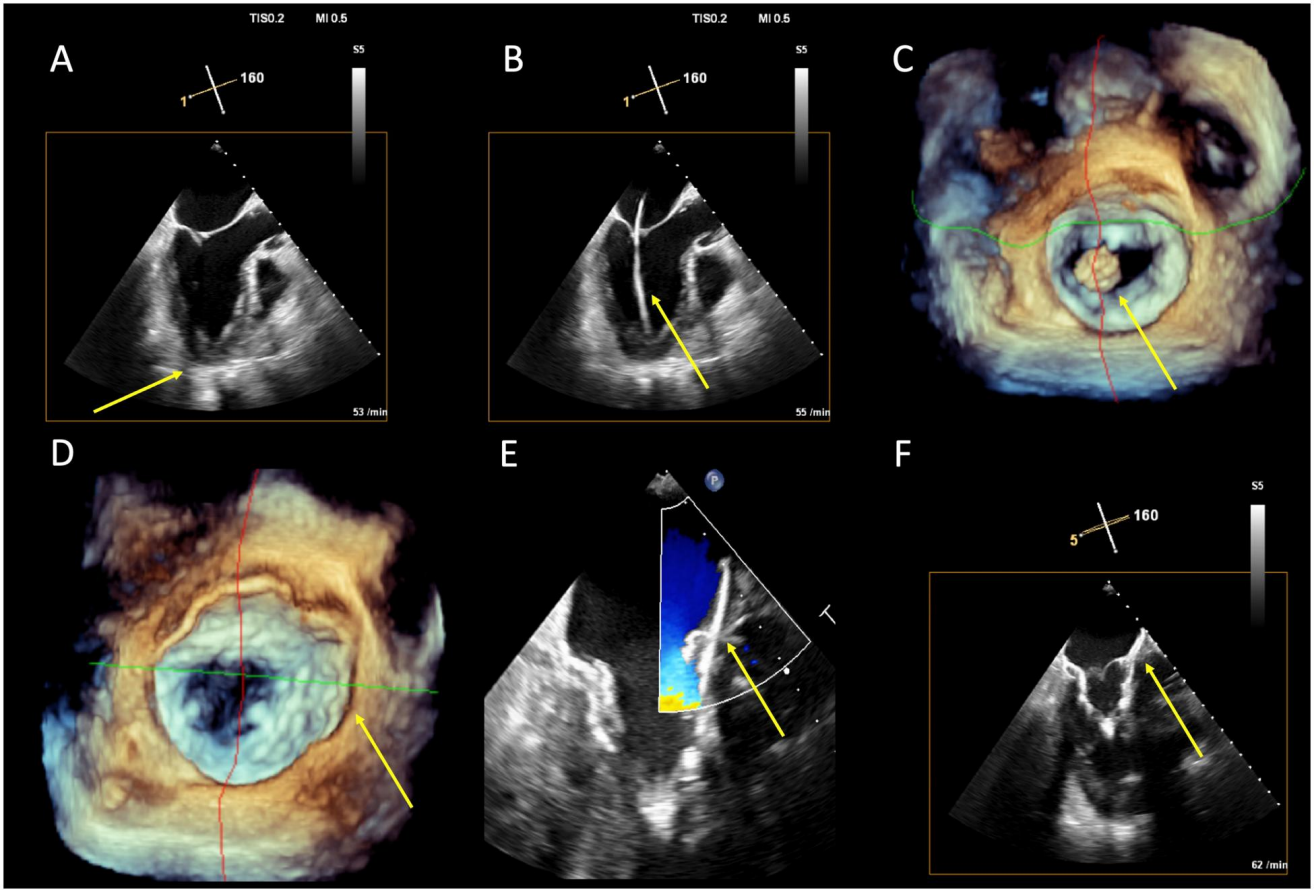
Procedural Steps and Echocardiographic Guiding of Transapical TMVI. (A) After exposure of the cardiac apex, transapical puncture is guided using bi-plane mode to display a bi-commissural and a 3-cavity (or LVOT) view. The arrow indicates the surgeon’s finger (poke test) in the 3-cavity view. (B) The implant catheter (arrow) is advanced over the standard guidewire through the valve and its position optimized using 2 reference views (here only 3-cavity shown), so that it is centred 1 to 2 cm above the mitral annulus. (C) The Tendyne valve (arrow) is mechanically advanced through the catheter under constant view. The prosthesis is exposed in the atrium and optimal rotation is confirmed. (D) The Tendyne valve is deployed and self-expands (arrow points at the outer border of the stent frame). (E) Once the prosthesis has been deployed, the valve is assessed using colour Doppler from 0 to 180° to detect PVLs and, if necessary, an increase in traction on the tensioner can be applied as a corrective manoeuvre. (F) in the 3-cavity view and in the apical trans-gastric view (5-cavity equivalent), the patency of the neo-LVOT (indicated by the arrow) is assessed and the tension reduced in the event of significant obstruction

A bi-plane view is used during all the manipulations to fix the valve at the apex using the tensioner, to check that the tension applied to the valve is optimal so that it does not migrate into either the LA or the LV. When the valve is secured by the pad and the patient is haemodynamically stable, a final assessment of PVLs and neo-LVOT patency is made (**Figure 5**).

## POSTINTERVENTIONAL CONSIDERATIONS

Postoperative care is a key determinant of outcomes following TMVI. Immediate priorities include maintaining haemodynamic stability, preventing vasoplegia, and early detection of complications. A structured handover between procedural and postoperative teams ensures continuity of care.

Key postoperative priorities include:

- **Early extubation** in the operating room to reduce sedation risks and support rapid recovery;
- **Effective pain management** with multimodal analgesia (e.g., ESPB, non-opioid medications);
- **Haemodynamic monitoring** with guided fluid management and defined escalation criteria;
- **Early mobilization** on the day of the procedure to prevent pulmonary complications;
- **Prompt removal of drains and catheters** within 24 hours;
- **Telemetry monitoring and routine TTE** within 48 hours or earlier if needed.

Patients are admitted to a specialized intermediate care unit and typically transferred to a telemetry ward the next morning. Continuous pulmonary artery catheter monitoring is not routinely used. Standardized protocols focus on normothermia, adequate oxygenation, and early rehabilitation to support optimal recovery.

## MANAGEMENT OF COMPLICATIONS

The most frequent complications during and after transapical TMVI include (1) haemodynamic instability due to LVOT obstruction; (2) uncontrolled apex bleeding; (3) valve embolization into LA or LV; and (4) paravalvular leak potentially causing haemolysis. Due to potential rapid haemodynamic deterioration, options for mechanical circulatory support (MCS; compact cardiopulmonary support or primed heart-lung machine) should be available. Preparation of venous and artery access (also needed for measurement of the LV pressure) is advisable before transapical puncture.

Avoidance of LVOT obstruction mainly consists of prevention using valve simulation and measurement of the anticipated neo-LVOT. Patients with a neo-LVOT ≤220mm^2^ are not suitable for Tendyne implantation. Anterior leaflet modification using either the LAMPOON technique^19^ (from the transseptal access) or leaflet cutting^20^ have been described. Importantly, due to the covered stent frame of the Tendyne valve, these techniques do not allow to significantly increase the size of the neo-LVOT and are used in case of a long hypermobile anterior leaflet to prevent systolic anterior motion (SAM) leading to dynamic leaflet obstruction. In case of acute LVOT obstruction, several bailout measures should be considered including partial recapture and lateral rotation of the valve to further minimize its projection into the LVOT, rapid sheath retrieval to improve diastolic filling and aggressive volume substitution, and finally, if the above-mentioned measures fail, full valve retrieval. Depending on LVOT obstruction severity, MCS may be needed to safely perform these steps and should be installed liberally to avoid uncontrollable haemodynamic deterioration. The use of pre-emptive (or rarely bailout) alcohol septal ablation has been also described.^21^^,^^22^

The rapid installation of MCS is key for the management of uncontrolled apex tearing and bleeding that can occur in fragile patients, those under immunosuppressive therapy (in particular steroids) and patients with ischaemic apex scaring and thinning. Importantly, MCS should not be withdrawn too early, but stay in place for 12 h-24 h to avoid recurrent bleeding due to re-exposure to the systemic blood pressures. Valve embolization usually requires valve retrieval using the dedicated tool. MCS may be needed to safely complete this step. This event was described to occur in about 4% of the commercially treated patients.^23^ Relevant PVL is a rare issued described in only 1.5% of the cases. Importantly, even non-severe PVL may lead to haemolysis and should be addressed during the procedure through optimization of valve placement and tensioning. In case of postprocedural migration, valve re-tensioning can be performed safely and reduces MR effectively in more than 91% of the patients.^18^ Successful treatment of relevant haemolysis using re- tensioning has also been described.^24^ The used of plugs is complicated by the atrial flange covering the LA ground but can also be attempted in selected cases.

## OUTCOMES AFTER TMVI

This review provides a comprehensive overview of the pre-, peri-, and postoperative management of patients undergoing TMVI with the Tendyne device, based on our interdisciplinary high-volume centre experience and current literature. Although the focus lies on the periprocedural phase, long-term outcomes are crucial to understanding the overall safety, efficacy, and patient benefit of TMVI.

Recent data support the durability and clinical benefit of the Tendyne device. The CHOICE-MI registry, which evaluated 10 dedicated TMVI systems including Tendyne, demonstrated effective and sustained MR elimination at 1 year, with residual MR ≤1 in 95.2% of patients. Despite a high-risk cohort, the combined end-point of all-cause mortality and heart failure hospitalization occurred in 39.2% of TMVI-treated patients, lower than in comparable high-risk surgical (42.9%) and conservatively treated (47.9%) populations.^25^

Similarly, Sorajja et al reported on the first 100 patients treated with the Tendyne system, showing MR elimination in 98.4% and a 1-year all-cause survival of 72.4%. Importantly, improvements in New York Heart Association (NYHA) functional class and symptom relief were also noted, indicating meaningful gains in quality of life and functional capacity, key components of patient-reported outcomes. These findings suggest that TMVI with the Tendyne prosthesis is not only technically effective but also provides tangible clinical benefits for symptomatic, inoperable patients with severe MR.^6^

## CONCLUSIONS

Multidisciplinary management is essential for the success of transapical transcatheter mitral valve implantation (TMVI), both during the procedure and throughout follow-up. Close collaboration between surgeons, anaesthesiologists, interventional cardiologists, and imaging specialists is critical to anticipate, prevent, and manage complications, particularly LVOT obstruction. Regular multidisciplinary planning meetings are recommended, especially in the early phase of program implementation and for anatomically complex cases at experienced centres.

Looking ahead, broader adoption of TMVI will depend on further refinements in patient selection, imaging protocols, and device technology. Long-term outcome data and patient-reported measures remain key areas for ongoing research. Future innovations, such as fully percutaneous approaches, improved delivery systems, and adaptive prosthesis designs, may expand the indications and improve outcomes in this high-risk population. Continued interdisciplinary collaboration and clinical research will be pivotal in advancing this field.

## Data Availability

The data underlying this article are available in the article.
